# *N*-Ethyl-*N*-(2-meth­oxy­phen­yl)benzene­sulfonamide

**DOI:** 10.1107/S1600536810023287

**Published:** 2010-06-23

**Authors:** Aziz-ur-Rehman, Humaira Rafique, Mehmet Akkurt, Nabila Dilber, Muhammad Athar Abbasi, Islam Ullah Khan

**Affiliations:** a Department of Chemistry, Government College University, Lahore 54000, Pakistan; b Department of Physics, Faculty of Arts and Sciences, Erciyes University, 38039 Kayseri, Turkey

## Abstract

In the title mol­ecule, C_15_H_17_NO_3_S, the C—S—N—C_benzene_ torsion angle is 81.45 (16)°, and the two aromatic rings form a dihedral angle of 45.83 (12)°. In the crystal structure, weak inter­molecular C—H⋯O hydrogen bonds link the mol­ecules into chains parallel to the *b* axis.

## Related literature

For the biological activity of sulfonamides, see: Ozbek *et al.* (2007 ▶); Parari *et al.* (2008 ▶). For related structures, see: Mariam *et al.* (2009 ▶); Arshad *et al.* (2009 ▶); Asiri *et al.* (2009 ▶); Khan *et al.* (2010 ▶); Akkurt *et al.* (2010*a* ▶,*b* ▶).
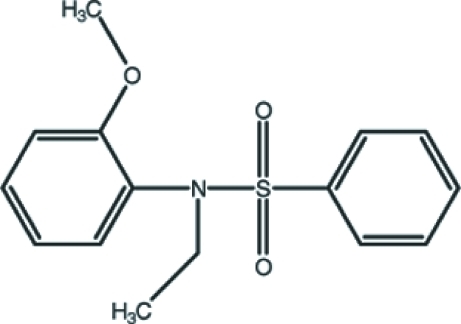


## Experimental

### 

#### Crystal data

C_15_H_17_NO_3_S
*M* *_r_* = 291.37
Monoclinic, 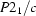

*a* = 9.3098 (5) Å
*b* = 9.5664 (6) Å
*c* = 17.1949 (10) Å
β = 104.040 (2)°
*V* = 1485.65 (15) Å^3^
*Z* = 4
Mo *K*α radiation
μ = 0.22 mm^−1^
*T* = 296 K
0.15 × 0.10 × 0.06 mm

#### Data collection

Bruker APEXII CCD diffractometer
13197 measured reflections
3670 independent reflections
1963 reflections with *I* > 2σ(*I*)
*R*_int_ = 0.045

#### Refinement

*R*[*F* ^2^ > 2σ(*F* ^2^)] = 0.051
*wR*(*F* ^2^) = 0.157
*S* = 0.99
3670 reflections
183 parameters
H-atom parameters constrained
Δρ_max_ = 0.30 e Å^−3^
Δρ_min_ = −0.27 e Å^−3^

### 

Data collection: *APEX2* (Bruker, 2007 ▶); cell refinement: *SAINT* (Bruker, 2007 ▶); data reduction: *SAINT*; program(s) used to solve structure: *SHELXS97* (Sheldrick, 2008 ▶); program(s) used to refine structure: *SHELXL97* (Sheldrick, 2008 ▶); molecular graphics: *ORTEP-3 for Windows* (Farrugia, 1997 ▶); software used to prepare material for publication: *WinGX* (Farrugia, 1999 ▶) and *PLATON* (Spek, 2009 ▶).

## Supplementary Material

Crystal structure: contains datablocks global, I. DOI: 10.1107/S1600536810023287/cv2735sup1.cif

Structure factors: contains datablocks I. DOI: 10.1107/S1600536810023287/cv2735Isup2.hkl

Additional supplementary materials:  crystallographic information; 3D view; checkCIF report

## Figures and Tables

**Table 1:** Hydrogen-bond geometry (Å, °)

*D*—H⋯*A*	*D*—H	H⋯*A*	*D*⋯*A*	*D*—H⋯*A*
C6—H6⋯O2^i^	0.93	2.53	3.300 (3)	140

Symmetry code: (i) 
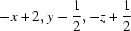
.

**Fig. 1.**
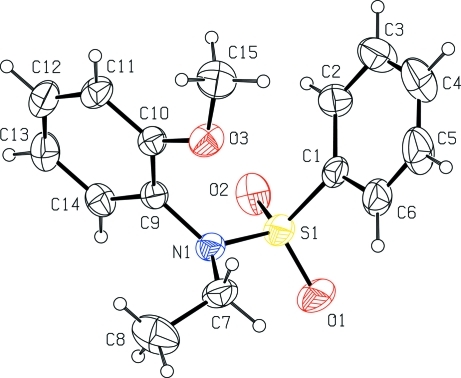
Molecular structure of (I) showing the atom labelling scheme, Displacement ellipsoids are drawn at the 30% probability level.

## supplementary crystallographic information

### Comment

Sulfonamides are known as biologically active compounds (Ozbek *et al.*, 2007; Parari *et al.*, 2008). As a contribution to a structural study of sulfonamide derivatives (Mariam *et al.*, 2009; Arshad *et al.*, 2009; Asiri *et al.*, 2009; Khan *et al.*, 2010; Akkurt *et al.*, 2010*a*,b) we present here the title compound, (I).

The title molecule (Fig. 1) is bent at the S atoms with the C1—S1—N1—C9 torsion angle of 81.45 (16)°. The dihedral angle between the phenyl (C1–C6) and benzene (C9–C14) rings is 45.83 (12)°.

In the crystal structure of (I), weak intermolecular C—H···O hydrogen bonds (Table 1) link the molecules into chains parallel to *b* axis.

### Experimental

A mixture of *N*-(2-methoxyphenyl)benzenesulfonamide (1.24 g, 5.0 mmol), sodium hydride (0.24 g, 10 mmol) and *N*,*N*-dimethylformamide (10 ml) was stirred at room temperature for 30 min and then ethyl iodide (0.4 ml, 5.0 mmol) was added. The stirring was continued further for a period of 3 h and the contents were poured over crushed ice. The precipitated product was isolated, washed and re-crystallized from methanolic solution. It was crystallized by slow evaporation of the solvent. Yield 72%.

### Refinement

All H atoms bonded to C atoms were positioned geometrically and refined using a riding model, with C—H = 0.93Å (aromatic), 0.96 Å (methyl) and 0.97 Å (methylene) with *U*_iso_(H) = 1.2*U*_eq_(aromatic, methylene) and *U*_iso_(H) = 1.5*U*_eq_(methyl).

### Crystal data

**Table:**

C_15_H_17_NO_3_S	*F*(000) = 616
*M**_r_* = 291.37	*D*_x_ = 1.303 Mg m^−^^3^
Monoclinic, *P*2_1_/*c*	Mo *K*α radiation, λ = 0.71073 Å
Hall symbol: -P 2ybc	Cell parameters from 3431 reflections
*a* = 9.3098 (5) Å	θ = 2.3–24.5°
*b* = 9.5664 (6) Å	µ = 0.22 mm^−^^1^
*c* = 17.1949 (10) Å	*T* = 296 K
β = 104.040 (2)°	Block, colourless
*V* = 1485.65 (15) Å^3^	0.15 × 0.10 × 0.06 mm
*Z* = 4	

### Data collection

**Table:**

Bruker APEXII CCD diffractometer	1963 reflections with *I* > 2σ(*I*)
Radiation source: sealed tube	*R*_int_ = 0.045
graphite	θ_max_ = 28.3°, θ_min_ = 3.1°
φ and ω scans	*h* = −12→10
13197 measured reflections	*k* = −11→12
3670 independent reflections	*l* = −20→22

### Refinement

**Table:**

Refinement on *F*^2^	Primary atom site location: structure-invariant direct methods
Least-squares matrix: full	Secondary atom site location: difference Fourier map
*R*[*F*^2^ > 2σ(*F*^2^)] = 0.051	Hydrogen site location: inferred from neighbouring sites
*wR*(*F*^2^) = 0.157	H-atom parameters constrained
*S* = 0.99	*w* = 1/[σ^2^(*F*_o_^2^) + (0.0842*P*)^2^] where *P* = (*F*_o_^2^ + 2*F*_c_^2^)/3
3670 reflections	(Δ/σ)_max_ < 0.001
183 parameters	Δρ_max_ = 0.30 e Å^−^^3^
0 restraints	Δρ_min_ = −0.27 e Å^−^^3^

### Special details

**Table:**

Geometry. Bond distances, angles *etc*. have been calculated using the rounded fractional coordinates. All su's are estimated from the variances of the (full) variance-covariance matrix. The cell e.s.d.'s are taken into account in the estimation of distances, angles and torsion angles
Refinement. Refinement on *F*^2^ for ALL reflections except those flagged by the user for potential systematic errors. Weighted *R*-factors *wR* and all goodnesses of fit *S* are based on *F*^2^, conventional *R*-factors *R* are based on *F*, with *F* set to zero for negative *F*^2^. The observed criterion of *F*^2^ > σ(*F*^2^) is used only for calculating -*R*-factor-obs *etc*. and is not relevant to the choice of reflections for refinement. *R*-factors based on *F*^2^ are statistically about twice as large as those based on *F*, and *R*-factors based on ALL data will be even larger.

### Fractional atomic coordinates and isotropic or equivalent isotropic displacement parameters (Å^2^)

**Table:**

	*x*	*y*	*z*	*U*_iso_*/*U*_eq_	
S1	0.86267 (6)	0.81474 (6)	0.18075 (4)	0.0619 (2)	
O1	0.99631 (18)	0.7591 (2)	0.16733 (12)	0.0964 (8)	
O2	0.81194 (19)	0.94809 (17)	0.14864 (9)	0.0771 (7)	
O3	0.57342 (18)	0.66902 (16)	0.25621 (9)	0.0675 (6)	
N1	0.73281 (18)	0.70249 (17)	0.14324 (10)	0.0532 (6)	
C1	0.8828 (2)	0.8200 (2)	0.28471 (13)	0.0557 (7)	
C2	0.8216 (3)	0.9285 (2)	0.31844 (14)	0.0687 (9)	
C3	0.8335 (3)	0.9308 (3)	0.39875 (16)	0.0899 (11)	
C4	0.9055 (4)	0.8251 (4)	0.44612 (18)	0.1036 (15)	
C5	0.9646 (4)	0.7163 (4)	0.4127 (2)	0.1047 (14)	
C6	0.9558 (3)	0.7136 (3)	0.33214 (17)	0.0809 (10)	
C7	0.7640 (3)	0.5514 (2)	0.15704 (15)	0.0723 (9)	
C8	0.6825 (4)	0.4652 (3)	0.09012 (17)	0.1080 (13)	
C9	0.5821 (2)	0.7500 (2)	0.12968 (11)	0.0476 (7)	
C10	0.5011 (2)	0.7321 (2)	0.18716 (12)	0.0498 (7)	
C11	0.3558 (2)	0.7784 (2)	0.17143 (14)	0.0633 (9)	
C12	0.2933 (3)	0.8395 (2)	0.09883 (17)	0.0740 (10)	
C13	0.3707 (3)	0.8566 (2)	0.04225 (15)	0.0722 (9)	
C14	0.5157 (3)	0.8111 (2)	0.05732 (13)	0.0605 (8)	
C15	0.5112 (3)	0.6721 (3)	0.32343 (15)	0.0878 (11)	
H2	0.77210	1.00010	0.28620	0.0820*	
H3	0.79260	1.00430	0.42150	0.1080*	
H4	0.91440	0.82730	0.50120	0.1240*	
H5	1.01110	0.64350	0.44500	0.1260*	
H6	0.99860	0.64090	0.30970	0.0970*	
H7A	0.86930	0.53530	0.16450	0.0870*	
H7B	0.73710	0.52310	0.20580	0.0870*	
H8A	0.57950	0.46310	0.09050	0.1620*	
H8B	0.72140	0.37180	0.09570	0.1620*	
H8C	0.69320	0.50430	0.04040	0.1620*	
H11	0.30120	0.76820	0.20970	0.0760*	
H12	0.19550	0.86980	0.08830	0.0890*	
H13	0.32660	0.89860	−0.00640	0.0870*	
H14	0.56880	0.82170	0.01840	0.0730*	
H15A	0.47490	0.76430	0.32950	0.1320*	
H15B	0.58550	0.64750	0.37070	0.1320*	
H15C	0.43090	0.60650	0.31570	0.1320*	

### Atomic displacement parameters (Å^2^)

**Table:**

	*U*^11^	*U*^22^	*U*^33^	*U*^12^	*U*^13^	*U*^23^
S1	0.0444 (4)	0.0686 (4)	0.0765 (4)	−0.0128 (3)	0.0219 (3)	−0.0093 (3)
O1	0.0473 (10)	0.1239 (15)	0.1290 (16)	−0.0121 (10)	0.0426 (10)	−0.0311 (12)
O2	0.0798 (12)	0.0655 (11)	0.0836 (11)	−0.0281 (9)	0.0152 (8)	0.0088 (8)
O3	0.0664 (10)	0.0780 (11)	0.0630 (10)	0.0078 (8)	0.0252 (8)	0.0158 (7)
N1	0.0449 (10)	0.0534 (11)	0.0631 (11)	−0.0040 (8)	0.0167 (8)	−0.0081 (8)
C1	0.0362 (11)	0.0496 (12)	0.0761 (15)	−0.0024 (9)	0.0033 (10)	−0.0073 (10)
C2	0.0755 (17)	0.0528 (14)	0.0751 (16)	0.0057 (12)	0.0132 (12)	−0.0031 (11)
C3	0.111 (2)	0.0808 (19)	0.0776 (19)	0.0022 (17)	0.0224 (16)	−0.0168 (15)
C4	0.127 (3)	0.106 (3)	0.0640 (17)	−0.010 (2)	−0.0037 (17)	−0.0083 (16)
C5	0.110 (3)	0.089 (2)	0.087 (2)	0.0113 (18)	−0.0303 (18)	0.0097 (17)
C6	0.0691 (17)	0.0680 (16)	0.089 (2)	0.0152 (13)	−0.0131 (14)	−0.0081 (13)
C7	0.0600 (15)	0.0580 (15)	0.1008 (18)	0.0077 (11)	0.0233 (13)	−0.0162 (12)
C8	0.143 (3)	0.0672 (18)	0.122 (2)	−0.0313 (18)	0.048 (2)	−0.0282 (16)
C9	0.0446 (12)	0.0434 (11)	0.0553 (12)	−0.0076 (9)	0.0130 (9)	−0.0070 (9)
C10	0.0464 (12)	0.0459 (11)	0.0576 (12)	−0.0058 (9)	0.0135 (9)	−0.0012 (9)
C11	0.0455 (13)	0.0638 (14)	0.0839 (17)	−0.0052 (10)	0.0224 (11)	−0.0003 (12)
C12	0.0492 (14)	0.0662 (16)	0.099 (2)	0.0021 (11)	0.0035 (13)	0.0004 (13)
C13	0.0691 (18)	0.0655 (15)	0.0701 (16)	−0.0038 (12)	−0.0059 (13)	0.0066 (12)
C14	0.0645 (15)	0.0628 (14)	0.0537 (13)	−0.0128 (11)	0.0132 (11)	−0.0029 (10)
C15	0.093 (2)	0.110 (2)	0.0686 (17)	−0.0052 (17)	0.0353 (14)	0.0107 (14)

### Geometric parameters (Å, °)

**Table:**

S1—O1	1.4226 (19)	C12—C13	1.354 (4)
S1—O2	1.4243 (17)	C13—C14	1.382 (4)
S1—N1	1.6286 (18)	C2—H2	0.9300
S1—C1	1.752 (2)	C3—H3	0.9300
O3—C10	1.356 (2)	C4—H4	0.9300
O3—C15	1.413 (3)	C5—H5	0.9300
N1—C7	1.482 (3)	C6—H6	0.9300
N1—C9	1.439 (3)	C7—H7A	0.9700
C1—C2	1.378 (3)	C7—H7B	0.9700
C1—C6	1.376 (3)	C8—H8A	0.9600
C2—C3	1.359 (4)	C8—H8B	0.9600
C3—C4	1.368 (5)	C8—H8C	0.9600
C4—C5	1.367 (5)	C11—H11	0.9300
C5—C6	1.368 (4)	C12—H12	0.9300
C7—C8	1.468 (4)	C13—H13	0.9300
C9—C10	1.392 (3)	C14—H14	0.9300
C9—C14	1.378 (3)	C15—H15A	0.9600
C10—C11	1.386 (3)	C15—H15B	0.9600
C11—C12	1.373 (4)	C15—H15C	0.9600
			
O1···C11^i^	3.336 (3)	C11···H15A	2.6800
O1···C12^i^	3.347 (3)	C11···H15C	2.9200
O2···C6^ii^	3.300 (3)	C15···H11	2.5800
O2···C14	3.113 (3)	H2···O2	2.5300
O3···N1	2.734 (2)	H2···C11^v^	3.0800
O3···C2	3.383 (3)	H2···H15C^v^	2.4600
O3···C7	2.965 (3)	H3···H8C^viii^	2.4400
O3···C1	3.152 (3)	H4···O1^viii^	2.8900
O1···H11^i^	2.7600	H6···O1	2.6900
O1···H7A	2.4400	H6···O2^vi^	2.5300
O1···H4^iii^	2.8900	H7A···O1	2.4400
O1···H12^i^	2.7600	H7A···C1^vi^	3.0600
O1···H6	2.6900	H7A···C2^vi^	3.0000
O2···H13^iv^	2.8800	H7B···O3	2.3800
O2···H15C^v^	2.9100	H7B···C10	2.9300
O2···H2	2.5300	H8A···C9	2.8200
O2···H6^ii^	2.5300	H8A···H15A^ix^	2.4700
O3···H7B	2.3800	H8C···C3^iii^	3.0900
N1···O3	2.734 (2)	H8C···H3^iii^	2.4400
C1···O3	3.152 (3)	H11···O1^vii^	2.7600
C2···O3	3.383 (3)	H11···C15	2.5800
C6···O2^vi^	3.300 (3)	H11···H15A	2.2900
C6···C7	3.472 (4)	H11···H15C	2.4700
C7···C6	3.472 (4)	H12···O1^vii^	2.7600
C7···O3	2.965 (3)	H13···O2^iv^	2.8800
C11···O1^vii^	3.336 (3)	H14···H15B^iii^	2.6000
C12···O1^vii^	3.347 (3)	H15A···C11	2.6800
C14···O2	3.113 (3)	H15A···H11	2.2900
C1···H7A^ii^	3.0600	H15A···C8^v^	2.9600
C2···H7A^ii^	3.0000	H15A···H8A^v^	2.4700
C3···H8C^viii^	3.0900	H15B···H14^viii^	2.6000
C8···H15A^ix^	2.9600	H15C···C11	2.9200
C9···H8A	2.8200	H15C···H11	2.4700
C10···H7B	2.9300	H15C···O2^ix^	2.9100
C11···H2^ix^	3.0800	H15C···H2^ix^	2.4600
			
O1—S1—O2	119.54 (11)	C4—C3—H3	120.00
O1—S1—N1	106.47 (10)	C3—C4—H4	120.00
O1—S1—C1	107.15 (11)	C5—C4—H4	120.00
O2—S1—N1	107.01 (10)	C4—C5—H5	120.00
O2—S1—C1	108.24 (10)	C6—C5—H5	120.00
N1—S1—C1	107.96 (9)	C1—C6—H6	120.00
C10—O3—C15	119.46 (18)	C5—C6—H6	120.00
S1—N1—C7	118.81 (15)	N1—C7—H7A	109.00
S1—N1—C9	117.18 (13)	N1—C7—H7B	109.00
C7—N1—C9	118.65 (18)	C8—C7—H7A	109.00
S1—C1—C2	119.88 (16)	C8—C7—H7B	109.00
S1—C1—C6	119.90 (18)	H7A—C7—H7B	108.00
C2—C1—C6	120.2 (2)	C7—C8—H8A	109.00
C1—C2—C3	120.0 (2)	C7—C8—H8B	109.00
C2—C3—C4	120.1 (3)	C7—C8—H8C	109.00
C3—C4—C5	120.1 (3)	H8A—C8—H8B	109.00
C4—C5—C6	120.6 (3)	H8A—C8—H8C	109.00
C1—C6—C5	119.1 (3)	H8B—C8—H8C	109.00
N1—C7—C8	112.2 (2)	C10—C11—H11	120.00
N1—C9—C10	121.41 (17)	C12—C11—H11	120.00
N1—C9—C14	119.11 (19)	C11—C12—H12	119.00
C10—C9—C14	119.5 (2)	C13—C12—H12	119.00
O3—C10—C9	115.92 (17)	C12—C13—H13	120.00
O3—C10—C11	124.52 (19)	C14—C13—H13	120.00
C9—C10—C11	119.56 (19)	C9—C14—H14	120.00
C10—C11—C12	119.4 (2)	C13—C14—H14	120.00
C11—C12—C13	121.5 (2)	O3—C15—H15A	109.00
C12—C13—C14	119.6 (2)	O3—C15—H15B	109.00
C9—C14—C13	120.5 (2)	O3—C15—H15C	109.00
C1—C2—H2	120.00	H15A—C15—H15B	109.00
C3—C2—H2	120.00	H15A—C15—H15C	109.00
C2—C3—H3	120.00	H15B—C15—H15C	109.00
			
O1—S1—N1—C7	42.39 (19)	S1—C1—C2—C3	178.2 (2)
O1—S1—N1—C9	−163.78 (15)	C6—C1—C2—C3	0.1 (4)
O2—S1—N1—C7	171.30 (16)	S1—C1—C6—C5	−177.1 (2)
O2—S1—N1—C9	−34.87 (17)	C2—C1—C6—C5	1.0 (4)
C1—S1—N1—C7	−72.39 (18)	C1—C2—C3—C4	−0.4 (4)
C1—S1—N1—C9	81.45 (16)	C2—C3—C4—C5	−0.5 (5)
O1—S1—C1—C2	144.01 (19)	C3—C4—C5—C6	1.7 (6)
O1—S1—C1—C6	−38.0 (2)	C4—C5—C6—C1	−1.9 (5)
O2—S1—C1—C2	13.9 (2)	N1—C9—C10—O3	0.7 (3)
O2—S1—C1—C6	−168.12 (19)	N1—C9—C10—C11	−179.48 (18)
N1—S1—C1—C2	−101.66 (19)	C14—C9—C10—O3	178.90 (18)
N1—S1—C1—C6	76.4 (2)	C14—C9—C10—C11	−1.2 (3)
C15—O3—C10—C9	167.2 (2)	N1—C9—C14—C13	179.41 (18)
C15—O3—C10—C11	−12.7 (3)	C10—C9—C14—C13	1.1 (3)
S1—N1—C7—C8	−148.4 (2)	O3—C10—C11—C12	−179.24 (19)
C9—N1—C7—C8	58.1 (3)	C9—C10—C11—C12	0.9 (3)
S1—N1—C9—C10	−93.0 (2)	C10—C11—C12—C13	−0.5 (3)
S1—N1—C9—C14	88.8 (2)	C11—C12—C13—C14	0.4 (3)
C7—N1—C9—C10	60.9 (3)	C12—C13—C14—C9	−0.7 (3)
C7—N1—C9—C14	−117.4 (2)		

Symmetry codes: (i) *x*+1, *y*, *z*; (ii) −*x*+2, *y*+1/2, −*z*+1/2; (iii) *x*, −*y*+3/2, *z*−1/2; (iv) −*x*+1, −*y*+2, −*z*; (v) −*x*+1, *y*+1/2, −*z*+1/2; (vi) −*x*+2, *y*−1/2, −*z*+1/2; (vii) *x*−1, *y*, *z*; (viii) *x*, −*y*+3/2, *z*+1/2; (ix) −*x*+1, *y*−1/2, −*z*+1/2.

### Hydrogen-bond geometry (Å, °)

**Table:**

*D*—H···*A*	*D*—H	H···*A*	*D*···*A*	*D*—H···*A*
C2—H2···O2	0.93	2.53	2.905 (3)	104
C6—H6···O2^vi^	0.93	2.53	3.300 (3)	140
C7—H7A···O1	0.97	2.44	2.911 (3)	109
C7—H7B···O3	0.97	2.38	2.965 (3)	118

Symmetry codes: (vi) −*x*+2, *y*−1/2, −*z*+1/2.
